# PWCDA: Path Weighted Method for Predicting circRNA-Disease Associations

**DOI:** 10.3390/ijms19113410

**Published:** 2018-10-31

**Authors:** Xiujuan Lei, Zengqiang Fang, Luonan Chen, Fang-Xiang Wu

**Affiliations:** 1 School of Computer Science, Shaanxi Normal University, Xi’an 710119, China; xjlei@snnu.edu.cn (X.L.); fangzq@snnu.edu.cn (Z.F.); 2 Key Laboratory of Systems Biology, Center for Excellence in Molecular Cell Science, Institute of Biochemistry and Cell Biology, Shanghai Institutes for Biological Sciences, Chinese Academy of Sciences, Shanghai 200031, China; 3 Center for Excellence in Animal Evolution and Genetics, Chinese Academy of Sciences, Kunming 650223, China; 4 School of Life Science and Technology, Shanghai Tech University, Shanghai 201210, China; 5 Department of Mechanical Engineering and Division of Biomedical Engineering, University of Saskatchewan, Saskatoon, SK S7N 5A9, Canada

**Keywords:** circRNA-disease associations, pathway, heterogeneous network

## Abstract

CircRNAs have particular biological structure and have proven to play important roles in diseases. It is time-consuming and costly to identify circRNA-disease associations by biological experiments. Therefore, it is appealing to develop computational methods for predicting circRNA-disease associations. In this study, we propose a new computational path weighted method for predicting circRNA-disease associations. Firstly, we calculate the functional similarity scores of diseases based on disease-related gene annotations and the semantic similarity scores of circRNAs based on circRNA-related gene ontology, respectively. To address missing similarity scores of diseases and circRNAs, we calculate the Gaussian Interaction Profile (GIP) kernel similarity scores for diseases and circRNAs, respectively, based on the circRNA-disease associations downloaded from circR2Disease database (http://bioinfo.snnu.edu.cn/CircR2Disease/). Then, we integrate disease functional similarity scores and circRNA semantic similarity scores with their related GIP kernel similarity scores to construct a heterogeneous network made up of three sub-networks: disease similarity network, circRNA similarity network and circRNA-disease association network. Finally, we compute an association score for each circRNA-disease pair based on paths connecting them in the heterogeneous network to determine whether this circRNA-disease pair is associated. We adopt leave one out cross validation (LOOCV) and five-fold cross validations to evaluate the performance of our proposed method. In addition, three common diseases, Breast Cancer, Gastric Cancer and Colorectal Cancer, are used for case studies. Experimental results illustrate the reliability and usefulness of our computational method in terms of different validation measures, which indicates PWCDA can effectively predict potential circRNA-disease associations.

## 1. Introduction

In recent years, an increasing number of circRNAs [1] have been uncovered and have drawn more attention than before. CircRNA is a newly discovered category of non-coding RNAs. Non-coding RNAs also include a large number of different RNAs, such as miRNAs, lncRNAs, piRNAs [2]. The first discovery of circular RNA was in the Tetrahymena cell [3]. There is an obvious difference between circular RNAs and common linear RNAs. That is, circRNA has a circular closed loop RNA structure, yet have no free 5’ and 3’ compared with linear RNAs [4]. In addition, circRNAs can also be classified into 4 categories as follows: Exonic circRNAs, intronic circRNAs, exonintron circRNAs and intergenic circRNAs [4,5]. Because of such a closed loop structures, they are usually stable, abundant, conserved, and tissue-specifically expressed [5].

With the progress of high throughput sequencing technology [6], more and more circRNAs have been confirmed to play significant roles in different biological processes [7]. According to many experiments, a large amount of circRNAs functions have been found to work as a scaffold in the assembly of protein complexes [8], and local subcellular positions [9], and so on. They also regulate the expression of their ancestor genes [10] and acts as a microRNA (miRNA) sponge [11,12]. Especially, many studies have proved that circRNA can be biomarkers of tumors [13,14,15].

Recently, a sharply increasing number of circRNAs have been discovered and there are also some circRNA-disease databases being developed, such as circR2Disease [16], Circ2Traits [17] and Circ2Disease [18]. Simultaneously, circRNAs-related diseases also have been verified by classic biological experiments. However, they are both time-consuming and expensive. Therefore, it is appealing to develop computational methods that can produce reliable prediction results and reduce both time and cost. Although, some computational methods have been proposed for predicting miRNA-disease associations [19,20,21], lncRNA-disease associations [22,23] and drug-target associations [18,24,25], there is no computational method for predicting circRNA-disease associations yet.

In this study, we propose the first computational method, Path Weighed method for predicting CircRNA-Disease Associations (PWCDA). After building a heterogeneous network consisting of three sub-networks, the disease similarity network, the circRNA similarity network and circRNA-disease association network, we calculate an association score for each circRNA-disease pair based on the paths connecting them in the heterogeneous network to determine whether a circRNA-disease pair is associated. Our method is evaluated with leave one out cross validation (LOOCV) and five-fold cross validation. The average AUC (Area Under roc Curve) of LOOCV is 0.900, while the AUC value of five-fold cross validation is 0.890. For further investigating the performance of our proposed model, we conduct several case studies of some common cancers. What’s more, we compare our method with some other computational prediction methods. The results show that our method outperforms other methods, which indicates that our proposed model has the better capability to predict potential circRNA-disease associations.

## 2. Results and Discussion

### 2.1. Effect of Parameter

Based on the previous study [26], we fix the maximum path length as 3. If the maximum path length is more than 3, not only do the running time of the method increases, but our method also takes some noisy information. In this study, we give a comprehensive analysis for the parameter α in our decaying function. After we calculate scores for each disease-circRNA pair, we can obtain a disease-circRNA association score matrix. Based on the scores matrix, we calculate the AUC. The results are represented in Table 1. It’s obvious that the effect of different values of α on the final AUC value is quite small and it can take value from 1 to 3. Therefore, we adopt the best result setting the value of α as 1. In order to reduce the running time, we don’t use any cross validation in this experiment. Furthermore, we also carry out an experiment to analyze another parameter, the threshold γ, which is represented in Table 2. For the sake of reducing the running time, any cross validation is not adopted. The result shows that the parameter γ might have tiny effect on the final AUC value. Thus, we set the γ value as 0.5, which gets the greatest AUC value.

### 2.2. LOOCV

**Figure 1:**
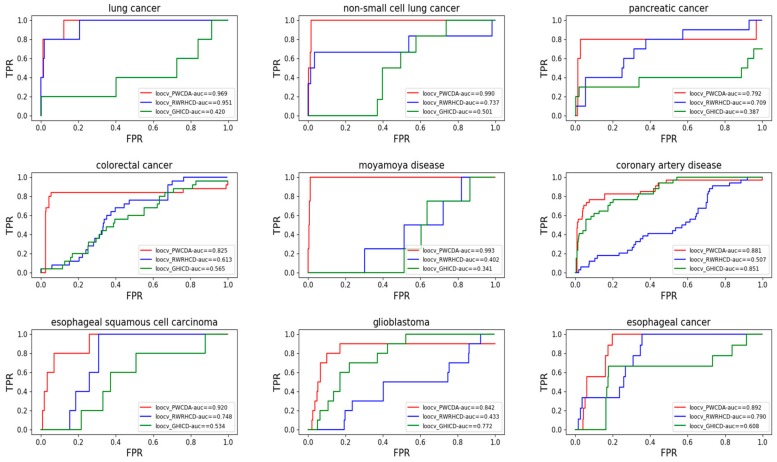
Comparison of Path Weighed method for predicting CircRNA-Disease Associations (PWCDA) with other models by leave one out cross validation (LOOCV). FPR, false positive rate.

For a given particular disease *i*, there are some associations between disease *i* and a number of circRNAs. In LOOCV, during each computational iteration, we leave one association out as a test data and use the remaining associations as a training dataset. If there is just one association between disease *i* and circRNAs in our dataset, we do not adopt LOOCV for this kind of disease. In LOOCV, we obtain an association score for each circRNA-disease pair and then rank all the prediction association scores. If a score value is greater than the pre-set threshold, we determine that the corresponding disease-circRNA is associated. With the change of the threshold, we can get a variety of true positive rates (TPRs) and false positive rates (FPRs), which can be used to draw the Receiver Operating Characteristic Curve (ROC) curve. In the end, we have compared our prediction method with other computational prediction methods [27,28]. The results can be found in Figure 1 and show that our proposed method outperforms the existing prediction methods.

### 2.3. Five-Fold Cross Validation

**Figure 2:**
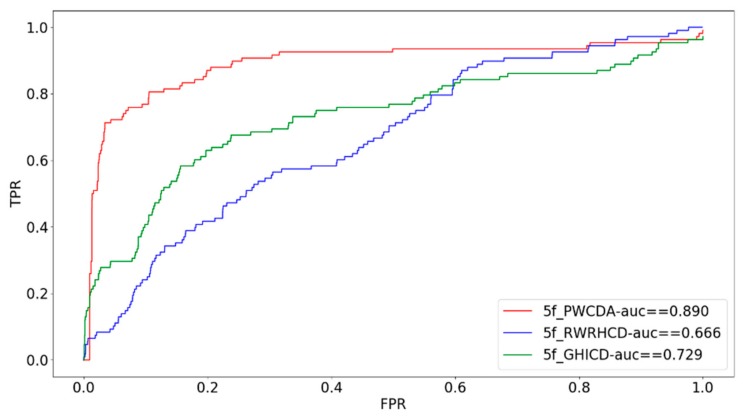
Comparison of PWCDA with other computational methods via five-fold cross validation.

In order to further illustrate the performance of our proposed method, we have adopted five-fold cross validation verification method as well for investigating the prediction performance. In our study, we divide all disease-circRNA associations into 5 parts. Each time we pick up one part as the test dataset and the remaining four parts consist of the training set. Then we can obtain the scores of all circRNA-disease associations. Similarly, we follow the same procedure as LOOCV to draw the AUC curve based on five-fold cross validation. What’s more, we have compared our proposed computational method with other prediction methods [27,28]. Our method gets more outstanding result than other methods, which is shown in the Figure 2.

### 2.4. Case Studies

Here, we also have conducted some case studies, which can help us further understand the associations between circRNAs and diseases. In this study, we choose three common diseases as prediction targets of our case studies, which are Breast Cancer [29], Gastric Cancer [30] and Colorectal Cancer [31]. In order to prove the prediction accuracy of our proposed method, we have used circRNA-disease database, and associations between circRNAs and diseases—which have been experimentally verified in the published articles [32].

Breast cancer is one the common cancers all over the world now [33], and breast cancer causes thousands of deaths every year. With the development of deep sequencing technology, circRNAs are confirmed to be biomarkers for diagnosing breast cancer. Based on our computational method, we have succeeded in predicting 29 of top 30 candidate circRNAs. For example, circpvt1 (top1) can be worked as miRNA spouse to regulate miRNA by moderating let-7 activity selected [30], and circRNA hsa_circ_104689 wasn’t predicted by our method and the predicting result have been presented in Table 3.

Gastric cancer [34] causes a high mortality rate in human. It can be produced in any tissue of the human stomach. These tumors in the stomach are usually malignant tumors, and they can also destroy the surrounding nervous tissue. With our computational method, there are 25 of top 30 candidate circRNAs that have been confirmed by another database, circRNA disease. For example, hsa_circ_0076304 (top1) and hsa_circ_0076305 (top2) are identified to downregulate in a group of gastric cancer [35]. circpvt1 (top3) can be regarded as the sponge of the miR-125 family [13], which can upregulate in the gastric cells. The more details of results are shown in Table 4.

Colorectal cancer [36] is one of the three most frequent cancers for women. Even though the incidence of colorectal cancer has been declined for a long time, a large proportion of patients die each year from colorectal cancer. In this study, we have succeeded in predicting 24 of top 30 candidate circRNAs. For example, hsa_circ_0001649 (top1) [31] has been identified to downregulate in colorectal cancer tissue. hsa_circ_0007534 (top2) [37] can upregulate in the different colorectal cancer cells. The more details of results are presented in Table 5.

## 3. Materials and Methods

### 3.1. Human circRNA-Disease Associations Network

All the circRNA-disease associations are downloaded from the website of circR2Disease database [16] (http://bioinfo.snnu.edu.cn/CircR2Disease/). This initial dataset contains 739 associations between 661 circRNA entities and 100 disease entities that are found based on three main species—human, mouse and rat. In this study, we select 541 circRNA entities and 83 human disease entities from our initial dataset, which includes Gastric cancer, Breast cancer, Colorectal cancer, etc. Finally, we obtain 592 circRNA-disease associations, which have experimentally been verified. These make up our circRNA-disease association network with adjacency matrix *M*. If there is a verified association between disease *i* and circRNA *j*, the entry *M*(*i*, *j*) is equal to 1, otherwise it is equal to 0.

### 3.2. CircRNA Semantic Similarity

For calculating circRNA semantic similarity, we download circRNA and its related gene targets dataset from circR2Disease. To measure circRNA semantic similarities, we also need to obtain gene related annotation terms that can be downloaded from Human Protein Reference Database (HPRD) database [38] (http://www.hprd.org/). Reviewing previous literature [39,40,41], there are some methods that can be referred to calculate the circRNA-related gene GO terms semantic similarities, including path-length-based methods, information-content-based methods, common-term-based methods and hybrid methods. In this study, we utilize a common-term-based method to measure circRNA similarity scores based on JACCARD index. In the previous studies [21,42], genes have been widely adopted to infer RNA similarity. Thus, the more gene related terms were shared by two circRNA *C_i_* and *C_j_*, the higher the similarity score they get. Denote *CS* as the circRNA semantic similarity matrix, and its entry *CS*(*i*, *j*) can be calculated by the following formula:

(1) $$CS(i,j)=\frac{\left|G_{i}\cap G_{j}\right|}{\left|G_{i}\cap G_{j}\right|}\;$$

where *G_i_*/*G_j_* denotes the GO terms that circRNA *C_i_*/*C_j_* target genes related.

### 3.3. Disease Functional Similarity

We adopt disease related gene annotations to measure disease functional similarities. These gene annotations are being extracted from two online databases. The first one is DisGeNET [43] (http://www.disgenet.org/web/DisGeNET/menu), which collects 381,056 gene-disease associations (GDAs) between 16,666 genes and 13,172 diseases. In addition, we also download disease phenotype data from OMIM [44]—Online Mendelian Inheritance in Man. OMIM is a biological database that is updated daily. We use the OMIM_2018_04_24 version. Then we integrate multiple annotation resources of diseases related genes, which help us get a more reliable performance.

There are also some methods for calculating disease similarities from previous studies[45]. The common methods include annotation-based measurements, function-based measurements and topology-based measurements [46,47,48,49]. We have adopted annotation-based methods to obtain disease similarities. We apply the JACCARD index, which is a standard method for computing similarities based on two collections of finite numbers of elements so as to estimate the similarity scores between diseases. Let *g_di_* be a collection of annotations of a gene associated with disease *d_i_*. We calculate the functional similarity score of two diseases *d_i_* and *d_j_* based on the JACCARD similarity coefficient score of *g_di_* and *g_di_*. Denote *DS* as the disease functional similarity matrix, then its entry *DS*(*i*, *j*) can be calculated by the following formula:

(2) $$DS(i,j)=\frac{\left|g_{d_{i}}\cap g_{d_{j}}\right|}{\left|g_{d_{i}}\cup g_{d_{j}}\right|}\;$$

We have constructed circRNA semantic similarity matrix based on their related GO terms and disease functional similarity based on its related annotating genes. However, one essential weakness that cannot be ignored is that the aforementioned similarity matrices are sparse, which indicates similarity of many pairs of diseases (or circRNAs) are unable to be calculated in their functional (or semantic) similarity matrices. To alleviate this weakness, the Gaussian interaction profile (GIP) kernel similarity [50,51] is adopted in this study to get additional information about the similarity of diseases and circRNAs.

### 3.4. CircRNA GIP Kernel Similarity

There is an assumption that the more similar the circRNA is, the more likely similar patterns of association and non-association with diseases. The GIP kernel similarity is adopted to calculate similarity based on the topological features of the known associations network widely, such miRNA-disease associations network [52], lncRNA-disease associations networks [53] and drug-target association network [54]. Accordingly, GIP kernel similarity is also used in this study to calculate the similarity of circRNA and disease. According to previous literature [54], we use a binary vector *C*(*i*) to indicate whether circRNA *i* is associated with diseases. The GIP kernel similarity between circRNA *C*(*i*) and *C*(*j*) can be computed by the following formula:

(3) $$KC(i,j)=exp(-\gamma_{c}{\left\|C(i)-C(j)\right\|}^{2})\;$$

To overcome the shortcomings that the disease functional similarity matrix and circRNA semantic matrix are sparse matrices, the parameter $\gamma_{c}$ is to adjust the kernel bandwidth, which can be calculated by the following formula:

(4) $$\gamma_{c}={\gamma^{\prime }}_{c}/\left(\frac{1}{n_{c}}\sum_{i}^{n_{c}}{\left\|C(i)\right\|}^{2}\right)\;$$

where *n_c_* is the number of circRNAs in our finial dataset. The parameter *γ*’*_c_* is set as 1 based on the previous study [54], which has obtained a better performance.

### 3.5. Disease GIP Kernel Similarity

We also calculate the GIP kernel similarity score between disease *i* and *j* as follows:

(5) $$KD(i,j)=exp(-\gamma_{d}{\left\|d(i)-d(j)\right\|}^{2}),$$

(6) $$\gamma_{d}={\gamma^{\prime }}_{d}/\left(\frac{1}{n_{d}}\sum_{i}^{n_{d}}{\left\|d(i)\right\|}^{2}\right),$$

where *d*(*i*) and *d*(*j*) are the association profiles of diseases *i* and *j*, respectively, *n_d_* is the number of diseases in our finial dataset, *γ*’*_d_* is also set to 1 based on previous studies.

### 3.6. Combine Multiple Similarity (circRNA and Disease)

We integrate the GIP kernel similarity for circRNAs with the semantic similarity of circRNAs to construct the circRNA similarity network. Specifically, the elements of the adjacency matrix of this network is calculated as follows:

(7) $$ICS(i,j)=\{\begin{array}{c} CS(i,j),\;if\;CS(i,j)\neq 0\\ KC(i,j),otherwise \end{array}.$$

We also integrate the GIP kernel similarity for diseases with the functional similarity diseases to construct the diseases similarity network. Specifically, the elements of the adjacency matrix of this network is calculated as follows:

(8) $$IDS(i,j)=\{\begin{array}{c} DS(i,j),\;if\;DS(i,j)\begin{array}{c} \neq 0 \end{array}\\ KD(i,j),otherwise \end{array}\;$$

### 3.7. Constructing Heterogeneous Network

After we obtain the final disease similarity scores and circRNA similarity scores. We can construct an initial heterogeneous network, which is composed of disease similarity network, circRNA network and disease-circRNA associations network.

In this initial heterogeneous network, there are some small weighted edges, which may represent noises. Therefore, to weaken the effect of those unimportant or noisy edges, we set a threshold *γ* (*γ* is equal to 0.5 based on previous studies [26] and our experiment) to remove them. Specifically, let *P_final_* and *P_initial_* be the adjacency matrices of the final and heterogeneous network, respectively, then we have:

(9) $$P_{final}(i,j)=\{\begin{array}{l} P_{initial}(i,j)\;P_{initial}(i,j)\geq \gamma \\ 0\;\mathrm{otherwise} \end{array}.$$

### 3.8. Perfomance Metrics

In this study, we adopt the AUC value to measure the prediction results. The AUC is the area under the ROC curve, which depicts the true positive rate (*TPR*) verse the false positive rate (*FPR*). The following equations are adopted to calculate the *TPR* and *FPR*:

(10) $$TPR=\frac{TP}{TP+FN}\;$$

(11) $$FPR=\frac{FP}{TN+FP}\;$$

where *TP* are positive samples (known associations), which are identified correctly, and *TN* are negative samples (unknown associations), which are identified correctly. *FP* are positive samples which are identified incorrectly while *FN* are negative samples, which are identified incorrectly.

### 3.9. PWCDA

**Figure 3:**
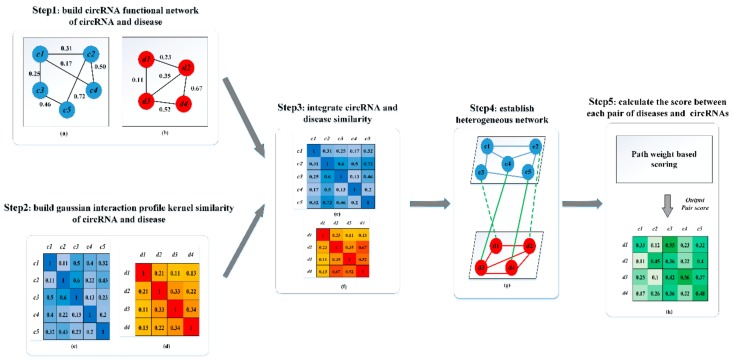
The flowchart of PWCDA is illustrated by five main steps. Step 1: Calculate circRNA semantic similarity and disease similarity scores, respectively. Step 2: Calculate GIP Kernel similarity scores for circRNAs and diseases. Step 3: Integrate circRNA (disease) semantic (functional) similarity with circRNA/disease GIP Kernel similarity, respectively. Step 4: Construct the heterogeneous network. Step 5: Calculate an association score for each circRNA-disease pair.

In this study, we proposed a novel computational model called PWCDA (a Path-Weighted CircRNA-Disease Associations method) to predict potential associations between circRNAs and diseases. The framework of our method is depicted in Figure 3. The computational method PWCDA traverses each node in each pathway without repeating based on heterogeneous network. To avoid traversing the same node repeatedly, we adopt the depth-first search (DFS) algorithm and mark the traversed nodes during each turn. Depth first search is implemented as a recursive function traversing the graph moving along the edge. We modify it to mark nodes, because they are accessed in recursion, and then delete tags before returning from recursive calls. In this study, we set the maximum searching length *η* as 3 steps according to previous studies [26], i.e., for circRNA *i* and disease *j*, there are several pathways, such as circRNA *i* connecting disease *j* directly, circRNA *i*’s neighbor circRNA connecting with disease *j* or circRNA *i* connecting with disease *j*’s neighbor diseases, circRNA *i*’s neighbor circRNAs connecting with disease *j*’s neighbor diseases directly. The choice of these paths is based on a hypothesis that the larger similarity score is between two circRNAs, the higher probability that they have the same associations is. Thus, after the weight of each circRNA-disease pair within all three paths are summed up. We can obtain the final scores between each circRNA-disease pair.

The more the number of paths between circRNA *j* and disease *i* exists, the greater the predictive score they obtain. Accordingly, the path set that connects circRNA *C_j_* to disease di can be represented as {*p*1, *p*2, …, *pm*}, where m is the number of the paths that connect disease *d_i_* and circRNA *C_j_* with the length less than *η*. The final predictive scores of *C_j_* and *d_i_* can be calculated as follows:

(12) $$score(d_{i},C_{j})=\sum_{k=1}^{m}{\left(S_{path}(p_{k})\right)}^{f_{weak}(len(p_{k}))}\;$$

where *S_path_*(*P_k_*) is the score of the path *p_k_* = {*e*_1_, *e*_2_, …, *e_n_*} [42] can be calculated as follows:

(13) $$S_{path}(p_{k})=\prod_{t=1}^{n}W_{e_{t}}\;(n\leq \eta )\;$$

The longer the path is, the smaller the contribution it is made, which means that the longer path would have less effect on predicting potential circRNA-disease associations than the shorter one. Therefore, the decaying function is an exponential function to reduce the influence of long path on final prediction scores, which can be represented as Equation (14):

(14) $$f_{weak}(len(p_{k}))=\alpha \times exp(len(p_{k}))\;$$

where *α* is a constraint factor and *len*(*p_k_*) is the length of path *p_k_*.

**Figure 4:**
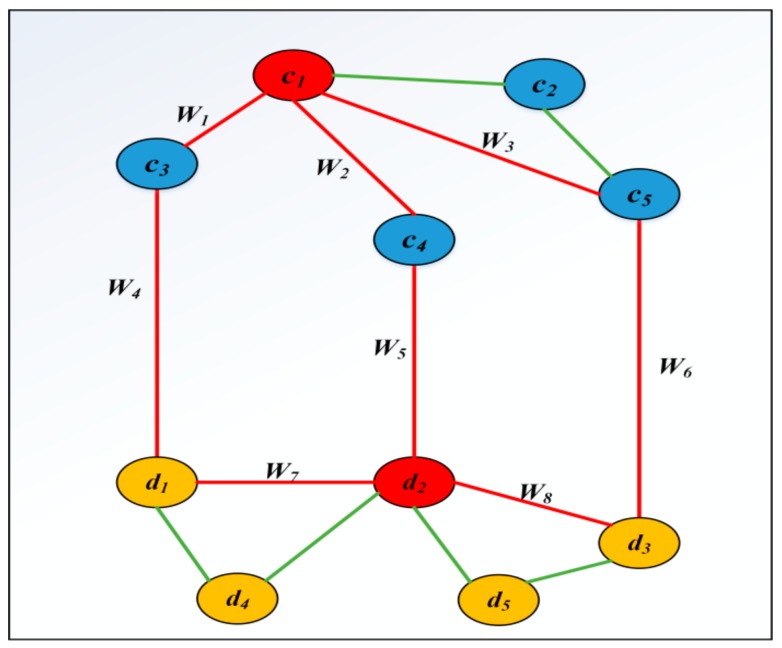
The path between *c_1_* and *d_2_* is within the maximum path length.

An example for calculating the score between circRNA *c_1_* and disease *d_2_* is shown in Figure 4. In the Figure 4, three paths {*c*_1_-*c*_4_-*d*_2_}, {*c*_1_-*c*_3_-*d*_1_-*d*_2_} and {*c*_1_-*c*_5_-*d*_3_-*d*_2_}, which are marked as red, are used to calculate the score between *c*_1_ and *d*_2_. Therefore, the score of *c*_1_ and *d*_2_ can be calculated as follows: Score (*c*_1_, *d*_2_) = {*c*_1_-*c*_4_-*d*_2_} (*w*_2_ × *w*_5_)^3*exp(2)^ + {*c*_1_-*c*_3_-*d*_1_-*d*_2_} (*w*_1_ × *w*_4_ × *w*_7_)^3*exp(3)^ + {*c*_1_-*c*_5_-*d*_3_-*d*_2_} (*w*_3_ × *w*_6_ × *w*_8_)^3*exp(3)^. There are also some other paths that can connect c_1_ with d_2_. Because the length of those paths, such as {*c*_1_-*c*_2_-*c*_5_-*d*_3_-*d*_2_}, are more than 3, we don’t consider this path.

## 4. Conclusions

With the increasing number of diseases related to circRNAs being discovered, more and more researchers have been paying attention to investigate diseases-related circRNAs. Although, experimental methods can find potential circRNA-disease associations with a high precision, the process is not only time-consuming, but also expensive. Here, we have proposed an effective computational method called PWCDA, which can predict potential circRNA-disease associations. Firstly, we calculate disease/circRNA similarities by combining their functional/semantic similarity and GIP kernel similarity. Secondly, we build a heterogeneous network, including the circRNA-disease association sub-network, the disease similarity sub-network and the circRNA similarity sub-network. PWCDA searches all the paths within three steps to compute an association score for each circRNA-disease pair to determine if a circRNA-disease pair is associated.

To thoroughly investigate the performance of our proposed method, we adopt LOOCV and five-fold cross validation. Furthermore, we have also compared our method with two state-of-the-art prediction methods. The comparison results illustrate that our methods work much better than other methods. The AUC value of five-fold cross validation is 0.884. Moreover, we apply our method to three diseases: Breast Cancer, Gastric Cancer, Colorectal Cancer for case studies.

There are several significant factors, which may explain why our proposed method can get a better performance than other computational models. Firstly, we have taken into account the sparsity of disease/circRNA similarity sub-networks. Thus, we have integrated disease functional similarity scores and circRNA semantic similarity scores with their corresponding GIP kernel similarity scores. Secondly, according to previous studies, we just use the paths within three steps, which can reduce the noisy information. Although we have combined different similarity scores, there is still some information unavailable. Therefore, we set a threshold to remove those edges whose weights are less than the predefined threshold.

Although we get a much better performance than other computational models, we can’t ignore the limitation. The prediction of associations between circRNAs and diseases is a relatively new research field, and the amount of data that we can use is limited. The ratio of positive samples to negative samples of circRNA-disease association is seriously unbalanced. To solve this problem, we may have two main solutions. One is that we can update the circRNA-disease database to obtain new data. The other is that we can extract the same number of positive samples as that of negative samples. Furthermore, our computational method tends to predict those circRNA-disease associations that are covered in the known associations’ dataset, and it just predicts fewer novel circRNA-disease associations. Thus, we will adopt more biological data to overcome this weakness. As a future topic, we can apply this work to the disease diagnosis based on network biomarkers [55,56,57] and disease prediction based on dynamic network biomarkers [58,59,60] in an accurate and reliable manner.

## Figures and Tables

**Table 1:** The Area Under roc Curve (AUC) value based on changing α and fixed pathway maximum length.

**α**	0.5	1	1.5	2	3	3.5	4	4.5	5
**AUC**	0.97100	0.97209	0.97206	0.97208	0.97202	0.97010	0.97010	0.97010	0.96879

**Table 2:** The AUC value based on changing γ and fixed pathway maximum length.

**γ**	0.1	0.2	0.3	0.4	0.5	0.6
**AUC**	0.96483	0.96483	0.96483	0.96500	0.97209	0.97205

**Table 3:** The top 30 breast cancer related candidates circRNAs.

Breast Cancer					
Rank	circRNA Name/id	Evidences	Rank	circRNA Name/id	Evidences
1	circpvt1/hsa_circ_0001821	PMID:279280058	16	hsa_circ_0001667	circRNAdisease
2	circ-foxo3	circRNAdisease	17	hsa_circ_0085495	circRNAdisease
3	hsa_circ_0001313/circccdc66	PMID:28249903	18	hsa_circ_0086241	circRNAdisease
4	hsa_circ_0007534	PMID:29593432	19	hsa_circ_0092276	circRNAdisease
5	hsa_circ_0000284/circhipk3	PMID:27050392	20	hsa_circ_0003838	circRNAdisease
6	hsa_circ_0011946	PMID:29593432	21	circvrk1	PMID:29221160
7	hsa_circ_0093869	PMID: 29593432	22	circbrip	PMID: 29221160
8	hsa_circ_0001982	circRNAdisease	23	circola	PMID: 29221160
9	hsa_circ_0001785	circRNAdisease	24	circetfa	PMID: 29221160
10	hsa_circ_0108942	circRNAdisease	25	circmed13	PMID: 29221160
11	hsa_circ_0068033	circRNAdisease	26	circbc111b	PMID:28739726
12	circamot11/hsa_circ_0004214	circRNAdisease	27	circdennd4c	circRNAdisease
13	hsa_circ_0006528	circRNAdisease	28	hsa_circ_103110/hsa_circ_0004771	circRNAdisease
14	hsa_circ_0002113	circRNAdisease	29	hsa_circ_104689/hsa_circ_0001824	unconfirmed
15	hsa_circ_0002874	circRNAdisease	30	hsa_circ_104821/hsa_circ_0001875	circRNAdisease

**Table 4:** The top 30 gastric cancer related candidates circRNAs.

Gastric Cancer					
Rank	circRNA Name/id	Evidences	Rank	circRNA Name/id	Evidences
1	hsa_circ_0076305	circRNAdisease	16	circma0138960/hsa-circma7690-15	circRNAdisease
2	hsa_circ_0076304	circRNAdisease	17	hsa_circ_0000181	circRNAdisease
3	circpvt1/hsa_circ_0001821	circRNAdisease	18	hsa_circ_0000745	circRNAdisease
4	hsa_circ_0001649	unconfirmed	19	hsa_circ_0085616	circRNAdisease
5	hsa_circ_0000284/circhipk3	unconfirmed	20	hsa_circ_0006127	circRNAdisease
6	hsa_circ_0014717	circRNAdisease	21	hsa_circ_0000026	circRNAdisease
7	cdr1as/cirs-7/hsa_circ_0001946	unconfirmed	22	hsa_circ_0000144	circRNAdisease
8	hsa_circ_0003195	circRNAdisease	23	hsa_circ_0032821	circRNAdisease
9	hsa_circ_0000520	circRNAdisease	24	hsa_circ_0005529	circRNAdisease
10	hsa_circ_0074362	circRNAdisease	25	hsa_circ_0061274	circRNAdisease
11	hsa_circ_0001017	circRNAdisease	26	hsa_circ_0005927	circRNAdisease
12	hsa_circ_0061276	circRNAdisease	27	hsa_circ_0092341	circRNAdisease
13	circ-zfr	unconfirmed	28	hsa_circ_0001561	unconfirmed
14	circma0047905/hsa_circ_0047905	circRNAdisease	29	circlarp4	circRNAdisease
15	circma0138960/hsa_circ_0138960	circRNAdisease	30	hsa_circ_0035431	circRNAdisease

**Table 5:** The top 30 colorectal cancer related candidates circRNAs.

Colorectal Cancer					
Rank	circRNA Name/id	Evidences	Rank	circRNA Name/id	Evidences
1	hsa_circ_0001649	PMID:29421663	16	has-circ_0006174	circRNAdisease
2	hsa_circ_0007534	PMID:29364478	17	hsa_circ_0008509	circRNAdisease
3	cdr1as/cirs-7/hsa_circ_0001946	circRNAdisease	18	hsa_circ_0084021	circRNAdisease
4	hsa_circ_0000284/circhipk3	PMID:27050392	19	circ_banp	circRNAdisease
5	hsa_circ_0001313/circccdc66	circRNAdisease	20	hsa_circrna_103809	circRNAdisease
6	ciritch/hsa_circ_0001141/hsa_circ_001763	unconfirmed	21	hsa_circrna_104700	circRNAdisease
7	hsa_circ_0014717	PMID:29571246	22	hsa_circ_0000069	circRNAdisease
8	hsa_circ_0000567	PMID:29333615	23	hsa_circ_001988/hsa_circ_0001451	circRNAdisease
9	hsa_circ_000984/hsa_circ_0001724	circRNAdisease	24	hsa_circ_0000677/hsa_circ_001569/circabcc	circRNAdisease
10	hsa_circ_0020397	circRNAdisease	25	circ_kldhc10/hsa_circ_0082333	PMID:26138677
11	hsa_circ_0007031	circRNAdisease	26	circ_stxbp51	unconfirmed
12	hsa_circ_0000504	circRNAdisease	27	circ-shkbp1	unconfirmed
13	hsa_circ_0007006	circRNAdisease	28	circ-fbxw7	unconfirmed
14	hsa_circ_0074930	circRNAdisease	29	hsa_circ_0046701	unconfirmed
15	hsa_circ_0048232	circRNAdisease	30	circttbk2/hsa_circ_0000594	unconfirmed

## Abbreviations

PWCDA	Path Weighed method, for predicting CircRNA-Disease Associations
LOOCV	leave one out cross validation
GIP	Gaussian Interaction Profile
GO	Gene Ontology
AUC	Area Under roc Curve
ROC	Receiver Operating Characteristic Curve
TPR	True Positive Rate
FPR	False Positive Rate
